# Constitutional methylation of the *MLH1* promoter: a case series including tumors not typically caused by Lynch Syndrome

**DOI:** 10.1038/s41431-026-02149-z

**Published:** 2026-06-03

**Authors:** Lise Graversen, Jannie Assenholt, Inge Søkilde Pedersen, Malene Pontoppidan Stoico, Henrik Hager, Charlotte Lautrup, Marianne Geilswijk, Daniel D. Buchanan, Finlay A. Macrae, Ingrid M. Winship, Mette Christiansen, Annabeth Høgh Petersen, Allan Højland, Lone Sunde

**Affiliations:** 1 Department of Clinical Genetics, Aarhus University Hospital, Aarhus, Denmark; 2 Department of Molecular Medicine, Aarhus University Hospital, Aarhus, Denmark; 3 Department of Molecular Diagnostics, Aalborg University Hospital, Aalborg, Denmark; 4 Department of Clinical Medicine, Aalborg University, Aalborg, Denmark; 5 Clinical Cancer Research Center, Aalborg University Hospital, Aalborg, Denmark; 6 Department of Pathology, Aarhus University Hospital, Aarhus, Denmark; 7 Colorectal Oncogenomics Group, Department of Clinical Pathology, The University of Melbourne, Parkville, Melbourne VIC Australia; 8 University of Melbourne Centre for Cancer Research, Victorian Comprehensive Cancer Centre, Parkville, Melbourne VIC Australia; 9 Genomic Medicine and Family Cancer Clinic, Royal Melbourne Hospital, Parkville, Melbourne VIC Australia; 10 Department of Medicine, The University of Melbourne, Parkville, Melbourne, VIC Australia; 11 Department of Clinical Genetics, Lillebaelt Hospital, Vejle, Denmark; 12 Department of Clinical Genetics, Aalborg University Hospital, Aalborg, Denmark

**Keywords:** Genetic testing, Preventive medicine

## Abstract

Constitutional methylation of the *MLH1* promoter is a rare cause of Lynch Syndrome likely to be overlooked in daily clinical practice, as *MLH1* methylation is common in sporadic tumors. We present four unrelated Danish and Australian patients with Lynch syndrome phenotypes and constitutional *MLH1* methylation including two patients with methylation levels indicating mosaicism. The clinical features, immunohistochemistry, *MLH1* promoter methylation status and genomic analysis of probands and family members are presented. The patients varied greatly in their clinical presentation and included multiple tumors, and a breast cancer and a dermal lipofibroma, tumors not classically related to Lynch syndrome. However, these patients are difficult to distinguish from other Lynch patients based on the clinical presentation. Awareness of this rare phenomenon and systematic testing of potential carriers is paramount to detect this condition and crucial to estimate the risk of cancer and optimize care for patients and their families.

## Introduction

Lynch syndrome (LS) is a heritable cancer predisposition syndrome associated with an increased risk of especially colorectal cancer (CRC) and endometrial cancer. LS is typically caused by heterozygosity for an inactivating variant in one of the mismatch repair (MMR) genes *MLH1, MSH2, MSH6*, or *PMS2* [1]. However, constitutional *MLH1* promoter methylation has been identified as the cause of LS in 5-10% of patients with LS related cancers with absence of MLH1 protein expression, but without a detectable germline MMR-gene pathogenic variant [2]. The phenotype caused by *MLH1* promoter methylation and germline *MLH1* variants are indistinguishable.

Constitutional *MLH1* methylation causes transcriptional silencing of the affected allele in normal tissue [3]. More than 100 patients with constitutional *MLH1* methylation have been described [4, 5] including several reported to be mosaics [5, 6]. The term “primary” constitutional *MLH1* methylation is used when no germline sequence alterations are present in the *MLH1* promoter region. In patients with “secondary” constitutional *MLH1* methylation, a cis-acting sequence variant underlies the methylation [7–9].

Somatic *MLH1* methylation is the most common cause of MMR-deficient (dMMR) cancers. About 60% of sporadic dMMR CRCs exhibit the *BRAF* V600E variant [10]. This variant results in an increased level of the transcription factor MafG that binds to the promoter of *MLH1* and other genes, causing methylation [11]. *BRAF* p.V600E is rarely found in LS associated CRCs, but has been reported both in tumors from patients with a germline sequence variant [12], and a few tumors from patients with constitutional *MLH1* methylation [13, 14].

The fact that *MLH1* methylation is being used as an indication that a tumor showing absence of immunostaining of MLH1, is caused by somatic inactivation of *MLH1* rather than a genetic predisposition causes a challenge in detecting LS due to constitutional *MLH1* promotor methylation, as tumors in these patients, by definition, show *MLH1* methylation.

Here we describe the phenotypes of four patients with primary constitutional *MLH1* promoter methylation, not published previously.

## Methods

Between 2010 and 2024, four unrelated patients not reported elsewhere were diagnosed with constitutional methylation of the *MLH1* promoter at Aarhus University Hospital, Denmark, Aalborg University Hospital, Denmark, or Genomic Medicine and Family Cancer Clinic, Royal Melbourne Hospital, Australia. Results from MMR immunohistochemistry (IHC), *MLH1* methylation analysis, and genomic analysis are shown in Table 1. Methods used in patients 1-3 are described below. Methods used in patient 4 have been described previously [15]. Informed consent for publishing from patients was obtained. For deceased patients the consent was obtained from offspring.

**Table 1:** Clinical and genetic characteristics of the four patients.

Patient	Tissue (age)	IHC		Neoplastic cells in tumour (%)	*MLH1* methylation (%)	*BRAF* p.V600E	DNA sequence variant
		MLH1/PMS2	MSH2/MSH6				
**1**	Non-cancer tissue, FFPE colon				2		0
	CRC (24)	MLH1-/PMS2-	Normal	NA	20	0	*MLH1* c.3 G > A
**2**	Non-cancer tissue, blood				20		0
	In situ carcinoma, cervix (35)	Normal	Normal	5	14		NA
	Breastcancer, IDC, sin (40)	MLH1-/NA	Normal	NA	NA		NA
	AC caecum (47)	MLH1-/PMS2-	Normal	NA	NA	0	NA
	AC descending colon (47)	MLH1-/PMS2-	Normal	50	33	0	NA
	Endometrial cancer (50)	MLH1-/NA	Normal	50	50		NA
	Breast, DCIS, dxt (52)	Normal	Normal	60	43		NA
	BCC (54)	Normal	Normal	70	15		NA
	BCC (54)	Normal	Normal	NA	NA		NA
**3**	Non-cancer tissue, colon				NA		0
	Non-cancer tissue, lymph node				34		NA
	Endometrial cancer (60)	MLH1-/PMS2-	Normal	NA	NA		NA
	AC ascending colon (61)	MLH1-/PMS2-	Normal	50	100	0	NA
	Dermal lipofibroma (66)	MLH1-/PMS2-	Normal		94		NA
	AC rectum (75)	MLH1-/PMS2-	Normal	60	65		NA
**4**	Non-cancer tissue, blood				40		0
	Endometrial cancer (47)	MLH1/PMS2-	Normal	NA	40		Yes

*AC* Adenocarcinoma, *BCC* Basal cell carcinoma, *DCIS* Ductal carcinoma in situ, *IDC* Invasive ductal carcinoma, *IHC* Immunohistochemistry, ‘-‘: Loss, NA: Not available, sin: Left side, dxt: Right side, ‘0’: Not detected.

### DNA extraction

DNA from a blood sample from patient 2 was extracted and used for genome sequencing with Illumina DNA PCR Free Library Prep and NovaSeq 6000 (Illumina). DNA from non-cancer tissue from patient 1 and 3 and cancer tissue from patient 1 was extracted and used for genome sequencing with TWIST Library Preparation EF Kit 2.0, TWIST Custom Panel (TWIST Bioscience) and NovaSeq 6000 (Illumina).

### Sequence analysis

Sequence alignment and variant calling for patients 1-3 were done using a GATK-pipeline and GRCh38; data were filtered to identify variants in the following genes (*APC, AXIN2, BMPR1A, EPCAM, MLH1, MLH3, MSH2, MSH3, MSH6, MUTYH, NTHL1, PMS2, POLD1, POLE, PTEN, RNF43, RPS20, SCG5, SMAD4, STK11, TP53)* using VarSeq (Golden Helix). In patient 2 *ATM, BARD1, BRCA1/2, BRIP1, CHEK2, PALB2, RAD51C/D* were also analyzed.

Variants were interpreted using databases hosting frequencies (gnomAD), databases hosting known variants (InSiGHT, ClinVar, LOVD, HGMD) and gene specific guidelines developed by gene expert panels (ClinGen InSiGHT Hereditary Colorectal Cancer/Polyposis Variant Curation Expert Panel Specifications to the ACMG/AMP Variant Interpretation Guidelines for MLH1 Version 1.0.0) and ACMG guidelines [16]. *MLH1* was screened in its entirety, including all exons, introns, and 5000 base pairs of the promoter region.

### *MLH1* methylation analysis

DNA from patients 1-3 were bisulfite treated using EZ-96 DNA Methylation-Direct™ MagPrep kit (Zymo Research) according to the manufacturer’s instructions. Methylation status was determined using the QX200™ Droplet Digital PCR system (Bio-Rad). See Supplementary. Thresholds were set using the Positive Droplet Calling (PodCall) algorithm [https://bioconductor.org/packages/PoDCall/]. The percentage of methylated *MLH1* was estimated using the 4plex control as the measure of total DNA [17].

## Results

### Patient 1

**Fig. 1:**
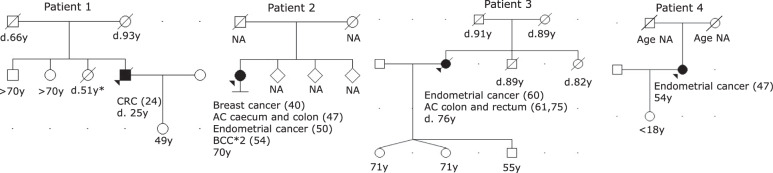
Pedigrees of the four families including cancer diagnoses. AC Adenocarcinoma, BCC Basal cell carcinoma, CRC Colorectal cancer, d. age of death, NA Not available, y Age in years, *Death not related to cancer.

In 1977, a 24-year-old Danish man was diagnosed with a tumor in the sigmoid colon. Lymph node metastases were present, and he died 18 months later. In 2017 his 40-year-old daughter sought genetic counselling. Analysis of tumor and normal tissue are shown in Table 1. There was no other family history of cancer (Fig. 1). DNA methylation analysis of the *MLH1* promoter and gene panel analysis on a blood sample from the daughter was normal.

### Patient 2

A now 64-year-old Danish woman was diagnosed with in situ squamous cell carcinoma of the uterine cervix age 35, invasive ductal carcinoma of the left breast age 40, mucinous adenocarcinoma of the caecum and mucinous adenocarcinoma of the descending colon age 47, and endometroid adenocarcinoma of the uterus age 50. At 52 years, in situ ductal carcinoma of the right breast was detected. At 54 years, she was diagnosed with two basal cell carcinomas on the scalp. Analyses of the patient’s tumors and blood are shown in Table 1. The pedigree showed no relatives with cancer (Fig. 1).

### Patient 3

A deceased Danish woman was diagnosed with endometroid adenocarcinoma of the endometrium age 60, adenocarcinoma of the ascending colon age 61, and mucinous adenocarcinoma of the rectum age 75. At the age of 66 she had a benign dermal lipofibroma removed. The patient died at the age of 76. There was no LS associated cancers in the family (Fig. 1). Analysis of the patient’s tumors and non-neoplastic tissue are shown in Table 1. Analysis of the *MLH1* promoter in DNA from blood from the three children showed no methylation.

### Patient 4

A 47-year-old Australian woman was diagnosed with endometrial cancer. There was no family history of cancer (Fig. 1). She had a young child who could not be subjected to testing. Analysis of the patient’s tumor and blood are shown in Table 1.

## Discussion

We present the clinical findings of four unrelated patients with constitutional methylation of the *MLH1* promoter identified in the normal daily practice of three different departments of clinical genetics without specific screening for this entity. Notably, two patients had multiple tumors including breast cancer and a dermal lipofibroma, tumors not classically regarded as LS tumors. We cannot exclude that ascertainment bias contributed to the high frequency of patients with multiple tumors. Moreover, an increased risk of tumors other than those regarded as “classical LS tumors”, such as breast cancer, has been observed in patients with LS [1].

We found the methylation percentage in non-cancer tissue to be 2, 20, 34 and 40, respectively. A methylation percentage of 2 indicates low-level constitutional methylation. It is likely that a low percentage of methylation in non-cancer tissue is associated with a lower risk. However, it is clear from the life course of patient 1 that this can still cause a very severe phenotype.

Methylation percentages in non-cancer tissue and cancer tissue with normal MLH1 expression by IHC in patient 2 also indicate mosaicism whereas the observed methylation percentages in patient 3 are more difficult to interpret. The methylation percentage for the tumor in the ascending colon of patient 3 was far above the expected when taking percentage of neoplastic cells into account. The analysis was repeated with the same result. Whether this is an artefact in the 35 years old formalin-fixed paraffin-embedded tissue or reflects a real biological phenomenon is unknown. The fraction of neoplastic nuclei could be higher than the estimated percentage of neoplastic cells, and the methylation level could be higher in the non-cancer colon tissue than the lymph node in patient 3. These are possible explanations for a methylation level higher than expected, even though they cannot fully explain an observed methylation level of 100% if there were a fraction of non-neoplastic cells in the sample.

We found no sequence variants in the MLH1 promoters of the four patients, and, thereby the cancer risk is not expected to follow an autosomal dominant pattern of inheritance. However, transmission of constitutional MLH1 methylation from mother to child, without any detectable sequence alteration, has been reported in two unrelated families [18, 19]. This warrants caution when evaluating the risk of close relatives of patients with apparently primary constitutional methylation.

Important limitations of this study include the age of some tissue samples and challenges in interpreting the methylation data. Comparison with methylation profiles from patients with germline MLH1 variants could improve interpretation.

Constitutional MLH1 methylation may be missed in patients with CRC or endometrial cancer if no systematic screening is done. The prevalence of constitutional MLH1 methylation is too low to justify testing of all patients with tumors with absence of MLH1 immunostaining due to MLH1 methylation and hence another strategy for identifying patients with a high risk of constitutional methylation is needed [20].

If patients with CRC/endometrial cancer with absence of MLH1 immunostaining and methylation of MLH1 diagnosed <50 years and patients with more than one tumor with MLH1 methylation at any age were tested for constitutional methylation, all the patients presented here would have been detected. An age threshold of ≤55 will reduce the risk of missing constitutional MLH1 methylation in patients with a milder clinical presentation [21].

## Conclusion

The predispositions caused by constitutional methylation may include a risk of tumors not traditionally regarded as LS related.

Awareness of this rare phenomenon and systematic testing is paramount to detect this condition.

## Supplementary information

Supplementary

## Data Availability

The data that support the findings of this study are available on request from the corresponding author. The data are not publicly available due to privacy or ethical restrictions.
