# Supplementary material for: Constitutional methylation of the MLH1 promoter: a case series including tumors not typically caused by Lynch Syndrome
Source: Eur J Hum Genet. 2026 Jun 3;34(8):1185–9. doi: 10.1038/s41431-026-02149-z (PMC13424574; doi:10.1038/s41431-026-02149-z)
Supplement: Supplementary file 1 — Supplementary [file 41431_2026_2149_MOESM1_ESM.docx]

**Supplementary**

**DNA methylation analysis using Droplet Digital PCR**

The QX200™ Droplet Digital PCR system (Bio-Rad) was used to determine the MLH1 promoter methylation status. The 22 µl ddPCR reaction consisted of 11 µl 2x ddPCR SuperMix for probes (no dUTP) (Bio-Rad), 818 nM of each primer, 182 nM of each probe and 5 µl bisulfite converted DNA (see table 1 for assay sequences). Droplets were generated using an Automated Droplet Generator (Bio-Rad) and PCR was performed on a CFX96 Touch Deep Well Real-Time PCR System (Bio-Rad) with the following conditions: 95°C for 10 minutes, 40 cycles of 94°C for 30 seconds and 60°C for 1 minute, and 98°C for 10 minutes with a ramp rate of 2°C/s. The plate was kept on hold at 4°C for 30 minutes before it was read by the QX200™ Droplet Reader (Bio-Rad). Each experiment included two methylation-positive controls (commercially available human methylated DNA (Zymo Research)), two methylation negative controls (commercially available human non-methylated DNA (Zymo Research)) and four no-template controls.

**Supplementary table 1**: Primer and probe sequences

| Assay name | Sense primer | Antisense primer | Probe | Ref |
| --- | --- | --- | --- | --- |
| MLH1 | GCGGATAGCG ATTTTTAACGC | CTTCGTCCCTC CCTAAAACGA | 6FAM-AGCGTATATTT TTTTAGGTAGCG-MGB | 2 |
| EPHA3 | GGATTTATTAGGTGTGTAATGTTATGGATT | ACTCCACATAAATCTTCTAAACTAAATTCCT | 6VIC-TTGGTTGAGAATAAATTGGGTTT-MGB | 1 |
| KBTBD4 | TTTGTATGTGGTGGGAGGGTTT | ACAAAAAAACACACCACTCCCAA | 6VIC-TATGTGGAAGTGTAATAATG-MGB | 1 |
| PLEKHF1 | GTAGTTTTAGATGGTTTTTTGAGTTGGA | CACTCCCATCCTATCTTCCCTCTATA | 6VIC-AGGGATTAGAGTAGGTTTG-MGB | 1 |
| SYT10 | GAGGTAAATGTAGGTTTTTAGTGTTGATTTT | CTTTATCCTCCCAATACTAATTATTATTTCTCC | 6VIC-AGTATGGGTATAGAATTTGT-MGB | 1 |

1. Pharo HD, Andresen K, Berg KCG, Lothe RA, Jeanmougin M, Lind GE. A robust internal control for high-precision DNA methylation analyses by droplet digital PCR. Clin Epigenetics. 2018;10:24.

2. Pinto D, Pinto C, Guerra J, Pinheiro M, Santos R, Vedeld HM, et al. Contribution of MLH1 constitutional methylation for Lynch syndrome diagnosis in patients with tumor MLH1 downregulation. Cancer Medicine. 2018;7(2):433-44.
